# Doubling the number of health graduates in Zambia: estimating feasibility and costs

**DOI:** 10.1186/1478-4491-8-22

**Published:** 2010-09-22

**Authors:** Aaron Tjoa, Margaret Kapihya, Miriam Libetwa, Joanne Lee, Charmaine Pattinson, Elizabeth McCarthy, Kate Schroder

**Affiliations:** 1 Clinton Health Access Initiative, Boston, USA; 2 The Ministry of Health, The Government of the Republic of Zambia, Lusaka, Zambia; 3 Clinton Health Access Initiative, Lusaka, Zambia

## Abstract

**Background:**

The Ministry of Health (MoH) in Zambia is operating with fewer than half of the human resources for health (HRH) necessary to meet basic population health needs. Responding urgently to address this HRH crisis, the MoH plans to double the annual number of health training graduates in the next five years to increase the supply of health workers. The feasibility and costs of achieving this initiative, however, are unclear.

**Methods:**

We determined the feasibility and costs of doubling training institution output through an individual school assessment framework. Assessment teams, comprised of four staff from the MoH and Clinton Health Access Initiative, visited all of Zambia's 39 public and private health training institutions from 17 April to 19 June 2008. Teams consulted with faculty and managers at each training institution to determine if student enrollment could double within five years; an operational planning exercise carried out with school staff determined the investments and additional operating costs necessary to achieve expansion. Cost assumptions were developed using historical cost data.

**Results:**

The individual school assessments affirmed the MoH's ability to double the graduate output of Zambia's public health training institutions. Lack of infrastructure was determined as a key bottleneck in achieving this increase while meeting national training quality standards. A total investment of US$ 58.8 million is required to meet expansion infrastructure needs, with US$ 35.0 million (59.5%) allocated to expanding student accommodation and US$ 23.8 million (40.5%) allocated to expanding teaching, studying, office, and dining space. The national number of teaching staff must increase by 363 (111% increase) over the next five years. The additional recurring costs, which include salaries for additional teachers and operating expenses for new students, are estimated at US$ 58.0 million over the five-year scale-up period. Total cost of expansion is estimated at US$ 116.8 million over five years.

**Conclusions:**

Historic underinvestment in training institutions has crippled Zambia's ability to meet expansion ambitions. There must be significant investments in infrastructure and faculty to meet quality standards while expanding training enrollment. Bottom-up planning can be used to translate national targets into costed implementation plans for expansion at each school.

## Background

Many resource-limited countries are facing the challenge of too few health workers to care for their population. Not enough doctors, nurses, clinical officers, midwives, medical assistants, and other key healthcare cadres are produced from training institutions to staff the health workforce [1-5]. Critical staffing shortages prevent these countries from delivering basic health services and meeting their health-related Millennium Development Goals [1,6-9].

In 2005, the Government of the Republic of Zambia Ministry of Health (MoH) estimated that it had fewer than half of the health staff necessary to deliver basic health services across the country, with even more acute shortages at rural clinics [10,11]. The Ministry of Health National Health Strategic Plan 2006 to 2010 provided several strategies to increase the size of the health workforce through the improvement of training, management, and retention; how these strategies could be implemented or which combination of strategies could increase the health workforce size enough to reach staffing targets was not determined [10]. An analysis of these strategies in late 2007 found that staffing targets would never be met without a significant increase in health training institution graduates [5]. In response, the Ministry of Health National Training Plan 2008 provided top-down targets for doubling the number of graduates during a five year period.

This study is based on the operational planning process commissioned by the National Training Plan 2008 to analyze how to double health training institution graduates in the next five years while meeting national training quality standards. We used a bottom-up approach to assess the costs and feasibility of doubling graduates at each of the 39 public and private health training institutions in Zambia, which run a total of 72 health degree, diploma, and certificate programs [12]. The operational planning process was led by the MoH, with the Clinton Health Access Initiative providing technical support in assessment design, implementation and analysis. The assessment's structure, objectives and methodology underwent a MoH approval process carried out by the Human Resources Task Group on Training, a committee chaired by the MoH Directorate of Human Resources and Administration and comprised of other government and partner stakeholders. Progress updates and the final report were then presented to the Human Resources Technical Working Group, whose approval of the final draft resulted in the dissemination of the National Training Operational Plan report and formal MoH adoption of the assessment's training institution-specific enrollment and infrastructure scale-up targets.

## Methods

### Data collection

Members of staff in each training institution were interviewed to determine the feasibility and costs of expansion using a standardized field questionnaire. The questionnaire followed a semi-structured interview format. Training institution staff members were asked to report on what scale-up student enrollment targets were feasible and were then led through an operational planning process to determine the key actions and investments necessary to accomplish these targets and the associated timeframe. These key actions included hiring faculty, building infrastructure, and procuring training materials. All associated one-time investments and additional annual recurring costs were mapped out for the next five years. A database was built using Access (Office, Microsoft; 2007) to collect and track responses to the training institution interview questions during the administration of the questionnaire.

Starting on 17 April 2008, two field teams carried out a three week pilot of the assessment. Each assessment team was comprised of a total of four individuals from the MoH Directorates of Human Resources and Planning and the Clinton Health Access Initiative. During this period, the teams assessed five training institutions that in total offered eleven health training programs. The schools were chosen for their variety of programs, public and private management, regional diversity, and size. After the pilot phase, the remaining 34 training institutions were assessed over the subsequent two months until 19 June 2008.

Costing information for all aspects of expansion was collected from the Ministry of Health Directorate of Planning and Development, which oversees funding of most public health training institutions, and from schools that recently completed construction projects. Discussions were held with the Ministry of Works and Supply to evaluate the construction tendering process and to propose a shortened tendering process for training institution infrastructure scale-up. Assumptions for teaching staff salaries and housing allowances for teachers were obtained from the Public Service Management Division. Per-student costs, which encompass all non-faculty school maintenance and operational costs, were collected from schools. A stable 3500 Zambia kwacha to one United States dollar (US$) exchange rate was assumed.

### Analysis

A model in Excel (Office, Microsoft; 2007) was developed to project the infrastructure and faculty needs for training institutions with three degree programs or less, which covered over 90% of all schools in Zambia. Faculty needs were broken down by the need for both tutors, who provide the didactic lectures in the schools, and clinical teachers, who provide hands-on instruction during skills labs and at practicum sites. The model determined the minimum number of faculty needed at a school by dividing the weekly classroom, laboratory and practicum hourly requirements for each semester of each program by the set number of hours that a tutor lectures in a week. The weekly classroom, laboratory and practicum hourly requirements were collected from the professional regulatory bodies, which mandate the curriculum that each school must provide, and refined based on input from training schools. The set number of hours a tutor can lecture in a week was determined during assessments and later validated by the quality standards set by professional regulatory bodies. To calculate infrastructure needs, the expected annual size of the student body at each training institution was multiplied by national student-to-training-infrastructure standard quality ratios.

The Chainama College of Health Sciences, Evelyn Hone College, and University of Zambia's School of Medicine, which offer more than seven health training programs each, had to be assessed without the Excel model due to the unique complexity of the operations at each school. The assessment determined resource needs through several consultations with each school's management and training program heads.

An Excel-based calculating tool converted the individual school scale-up needs into one-time and additional annual recurring costs. Standard cost assumptions were used for all health training institutions.

### Quality standards consensus

A National Quality Standards Consensus workshop was convened for the first time to set national minimum infrastructure quality standards for health training institutions. Participants included professional regulatory bodies, training experts, MoH policy officers, and training institution administrators. The workshop set minimum quality standard ratios such as the maximum number of students per dormitory room, classroom, skills-lab, practicum site, library, computer lab, and dining hall, in addition to the maximum number of faculty per staff office and house [13]. The national quality standard ratios for the number of students per tutor and clinical teacher were also reviewed. This provided the quality ratios used to analyze resource needs in this analysis.

### Operational plans

**Figure 1:**
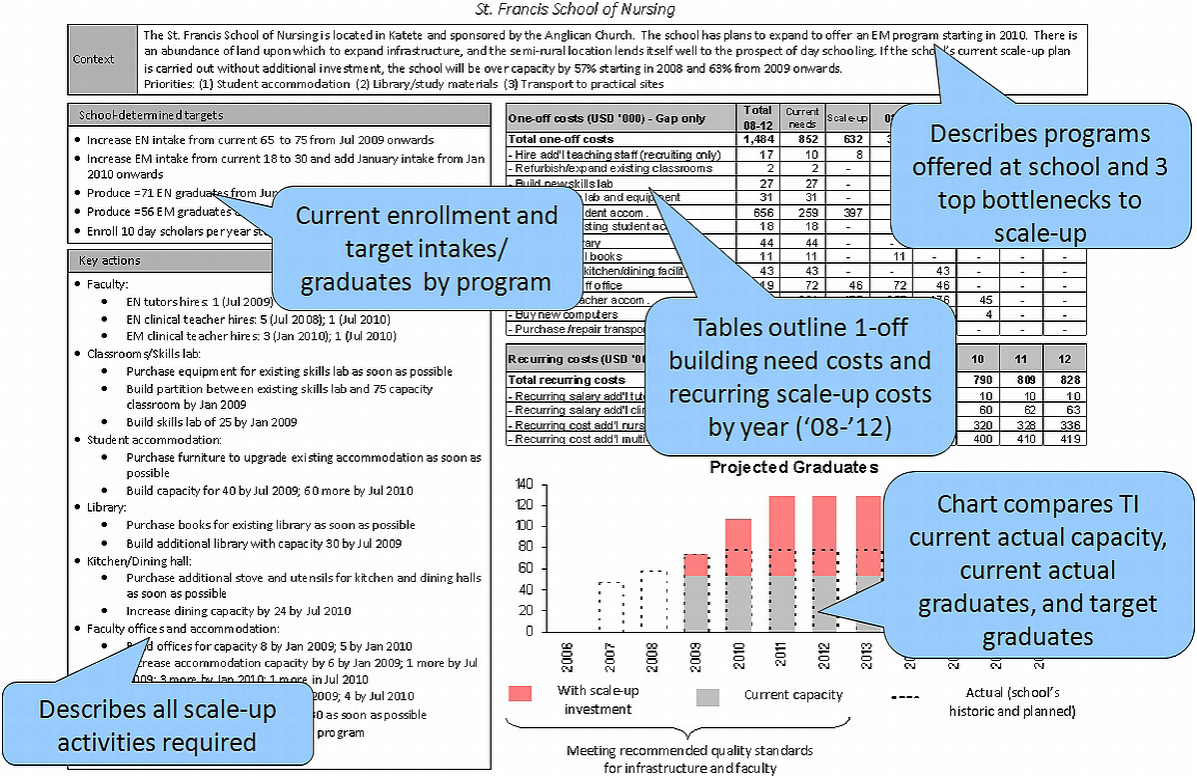
**Example school-specific one-page operational scale-up plan**. The study produced one-page summary plans that contained each school's annual student intake targets and the year in which the targets would be achieved. The key, prioritized actions necessary to accomplish these targets are listed along with target dates of completion. All associated one-time and additional recurring scale-up costs are detailed for five years. Lastly, the plans provide a description of current student capacity. The compilation of each school's one-page operational plan provided a national blueprint for the resources and activities needed to reach the National Training Plan targets.

School-specific operational plans were produced after the analysis and sent to each training institution for final review (see Fig. 1). The plans detailed each school's annual student targets and the year in which the targets would be completed. The key, prioritized actions necessary to accomplish these targets were listed along with target dates of completion. All associated one-time and additional recurring scale-up costs were detailed from 2008 to 2012. Lastly, the plans provided a description of the current student capacity.

## Results

### Publically operated schools

The individual school assessments determined that Zambia could increase aggregate annual health training enrollment at the 30 publically run schools by up to 94% over five years with sufficient financial support. This represents an aggregate increase in annual student intake at these schools from 1897 students in 2007 to 3675 students in 2012 (Table 1).

**Table 1:** National Training Plan 2008: current and target level of training output by cadre at public training institutions, Zambia

Cadre	Annual training institution output	
	
	2007 Output	2012 Output, Post Scale-up
Medical doctor	67	150

Nurse	900	1,636

Midwife	300	765

Medical licentiate	20	20

Clinical officer	133	190

Post basic nurse (teaching staff)	35	50

Laboratory staff	120	264

Pharmaceutical staff	120	150

Environmental health staff	96	240

Radiography staff	40	80

Paramedical staff	66	130

Total	1897	3675

The individual school assessments determined that Zambia could increase aggregate annual health training enrollment at the 30 public training institutions by up to 94% over five years with sufficient support, an increase from 1897 students in 2007 to 3675 students in 2012.

School administrators reported having a large enough pool of applicants to meet scale-up student intake targets without a loss in the quality of trainees. The annual applicant pools were described as having two to three times the number of qualified applicants as there were available slots for training. Qualified applicants were defined by administrators as applicants who had successfully completed the requisite basic certificate or diploma for each training program. Previously, schools preferred to accept candidates applying from their immediate geographical area to minimize the risk of attrition during the program. Dropout is low at most training schools (< 5%), with family issues or pregnancy as the leading cause of dropout. In increasing the size of their intakes, training institutions anticipated having to accept candidates from regions further from their school, which would present the risk of increasing the number of students that dropout because they have to return home for family reasons.

The expansion of publically run schools is estimated to cost US$ 116.8 million over five years. US$ 58.8 million of this total is needed for one-time infrastructure and furnishing costs, while US$ 58.0 million is needed for additional annual recurring costs. The additional annual recurring costs include expenditures for additional students (including food, training materials, and all non-faculty school maintenance and operational costs), faculty housing allowances, and new faculty salaries. The majority of the additional recurring costs are accounted for by the US$ 40.6 million (70.1%) needed for additional student costs (see Table 2). Faculty housing allowances, which total US$ 8.2 million across the five years, were provided in many cases instead of building faculty houses, since housing allowances were determined to be more cost-effective, with annual housing allowances costing only 8% of the cost of building a new house.

**Table 2:** Additional recurring costs needed to expand capacity at public training institutions by 94% over 5 years, Zambia

Additional annual recurring costs (USD '000)	2008	2009	2010	2011	2012	Total 08-12
Total additional recurring costs	3220	2976	11 327	17 389	23 038	57 950

- Recurring salary for additional tutors	457	668	1036	1391	1803	5355

- Recurring salary for additional clinical teachers	614	-	885	1005	1200	3704

- Faculty housing allowances	1566	-	1898	2184	2595	8244

- Recurring cost for additional students	583	2308	7507	12 809	17 439	40 647

Total training institution recurring costs are expected to increase each year due to the increase in the number of staff and students. This table details this by year and by cost category. Housing allowances are given to faculty where accommodation is not available and cost-effective to build. Many programs plan to increase their enrollment in 2010, accounting for smaller or no additional recurring costs in 2008 and 2009.

One-time investments in infrastructure are required both to expand capacity and to address immediate quality concerns. Nearly 70% of publically run schools were operating below recommended national infrastructure standards, resulting in overcrowding and reduced training quality. Nationally, student accommodation, staff office space, teacher accommodation, and library seating capacity must increase by over 150% from their current levels to ensure that training conditions meet recommended quality standards during scale-up (see Table 3).

**Table 3:** Overview of infrastructure expansion required to scale-up public training institutions by 94%, Zambia

Infrastructure	In 2007	In 2012, post scale-up
Beds for student accommodation	3101	8326

Classroom and lecture theatre seats	3967	7402

Library seats	744	2366

Skills, chemistry, biomedical lab seats	1218	1637

Staff office desks	259	707

Beds for teacher accommodation	48	146

There needs to be significant investments in order to achieve the 94% scale-up of training institutions. This table details the key infrastructure expansion needs to support scale-up.

For expansion to succeed, the number of tutors and clinical teachers must increase by 363 (111%). Broken down by teacher type, the aggregate number of tutors must increase from the current level of 260 to 431 (66% increase) in 2012 post scale-up. The need for clinical skills teachers was neither fully considered nor funded in the past, and to reach quality standards during scale-up, the aggregate number of clinical teachers must increase remarkably from 66 to 258 (291% increase).

Our costing of the expansion activities found that the most expensive one-time cost category is building, renovating, and furnishing student accommodation. This activity is estimated to cost US$ 35.0 million (59.5% of total one-time costs) across the 30 public training institutions (see Table 4). The remaining US$ 23.8 million (40.5% of total one-time costs) is spent on new staff offices, new staff accommodation (mostly in rural areas where renting is not feasible), new kitchen and dining hall facilities, new and refurbished classrooms and lecture theatres, new and refurbished libraries and books, and new chemical, biomedical, and skills laboratories.

**Table 4:** Total one-time costs that are necessary to achieve the 94% scale-up of public training institutions and meet quality training standards, Zambia

One-off costs (USD '000)	2008	2009	2010	2011	2012	Total
**Teaching**						

Hire additional teaching staff (recruiting costs only)	265	99	99	111	135	710

Build new classrooms	356	571	195	170		1292

Build new basic lecture theatres		556	158	908	310	1932

Refurbish or expand existing classrooms	42	146	6	2		197

Build new staff offices	2172	556	285	1145	475	4633

**Laboratories**						

Build new skills laboratories	118	120	84	29		350

Update current skills laboratories and equipment	160	425	31	64		681

Build new chemical/biomedical laboratories				115		115

Update chemical/biomed laboratories and equipment		308		215		523

**Student accommodation**						

Build new student accommodation	1679	9580	7824	9361	6344	34 788

Refurbish existing student accommodation	205	41				247

**Library**						

Build new libraries	154	1501	105	612	23	2395

Refurbish or expand existing libraries	6	37		16		59

Buy new text books	214	105	182	161	63	726

**Kitchen & dining**						

Build or update kitchen and dining facilities	472	1611	245	1857	8	4194

**Faculty accommodation**						

Build new teacher accommodation	3086	746	270	138		4239

**Computers**						

Buy new computers	57	78	65	92	47	339

**Vehicles**						

Purchase and repair transportation vehicles	367	235	215			817

**Other**						

Purchase teaching materials	4	2	1	0	0	7

Purchase recreational hall furniture	10	10	3			23

Build new sports complex		293	157			450

Build new guest house			90			90

**Total one-off costs**	**9 367**	**17 021**	**10 015**	**14 996**	**7407**	**58 807**


There need to be significant investments in order to achieve the 94% scale-up of public training institutions. This table displays the exact costs and year in which these investments are planned to occur. Construction costs are spread across the five years of scale-up.

Discussions with the Ministry of Works and Supply revealed that the time to commence a construction process during training institution expansion may be longer than the normal three to six month tendering process due to a current backlog of work and staff capacity constraints at the Ministry. While the tendering process cannot be shortened to accommodate the training institution expansion, this bottleneck could be addressed by hiring additional staff at the Ministry of Works and Supply. Alternatively, segments of the tendering process could be subcontracted to a consultancy firm, with approval and limited oversight from the Ministry of Works and Supplies.

### Privately operated schools

Annual health training enrollment at the 9 privately operated schools can scale-up from 507 students in 2007 to 830 by 2012. This will require an estimated investment of US$18 million in infrastructure costs over five years and hiring of 90 additional teaching staff, with 50 tutors and 40 clinical teachers. However, the assessment found that there were many quality improvements needed at many of these schools and recommended that these quality concerns be addressed prior to consideration of government investment in scaling-up capacity at these schools.

## Discussion

In line with the MoH's goal to significantly expand health worker training in Zambia, it was determined that training institution capacity at the publically operated schools could roughly double in the next five years. By visiting each of the individual training institutions in Zambia, we were able to validate the feasibility of this expansion target and to identify the minimum resources needed to achieve it. Since training institutions in Zambia have suffered from a lack of resources, large investments are needed both to attain basic quality training conditions and to expand training capacity. Physical training infrastructure must be built or renovated to create adequate training space for students, and more teaching faculty must be trained and retained to meet minimum teacher-to-student quality ratios.

Using the national training quality standards, we found that many schools were operating below the minimum infrastructure standards. Significant increases in infrastructure totaling US$ 58.8 million will be required in the next five years in order to scale-up and to meet these standards at publicly managed training institutions. Particular bottlenecks to expansion are student and faculty accommodation, in addition to library, dining, and office space. The three largest public multi-cadre training institutions faced the unique challenge of expanding their infrastructure in space-constrained, urban Lusaka. Each of these large schools addresses this space constraint through different expansion strategies, including renovations and add-ons to existing infrastructure, construction of off-campus housing, or relocation of the health training programs to a new campus.

The total infrastructure costs were spread across the five year scale-up period evenly, as construction had to be staggered over the five years of scale-up given the limited capacity of contractors, suppliers, construction staff, and materials available in Zambia. The estimated additional annual recurring costs rise steadily over the five year period, from US$ 3.2 million in 2008 to US$ 23.0 million in 2012.

Increases in faculty are necessary to achieve training institution expansion, with total need expanding steadily through 2012. We estimated that the number of teaching staff will need to roughly double. The government is working towards reaching this faculty staffing goal through expanding the training pipeline of faculty, including expanding the number of nursing bachelors and masters students graduating from the University of Zambia from 35 to 50 students annually and introducing a new direct-entry bachelors degree program for nurses. These trained nurses could then serve as tutors in nurse training institutions. Another policy that is being considered is the provision of housing and financial incentives to help retain faculty in rural areas. Similar retention schemes have proven effective at retaining healthcare staff in rural areas in Zambia, and therefore should have the potential to retain teaching faculty in rural areas [14]. The government is also considering hiring retired faculty. Finally, recognizing the successful use of technologies such as e-learning to reduce teaching staff needs in other countries, the training plan calls for the use of technology to teach students in Zambia, though no concrete initiatives have yet emerged [12,15].

Any rapid expansion of training faculty would be likely to include new faculty who would be teaching for the first time. The government and the professional regulatory bodies closely regulate the material taught in the classroom and set exact goals for the number of procedures that must be completed during practical training, and the new national training quality standards mandate specific teacher to student ratios. Therefore the content and the number of students covered by each teacher should remain constant. However, measures will need to be taken to ensure the quality of teaching of the new faculty.

Infrastructure costing assumptions were based on the costs incurred during the most recent training institution infrastructure expansion projects, which followed a standard design for infrastructure developed and approved by the MoH for training schools. We did not estimate the impact of market fluctuations on the cost of infrastructure, as the price of major cost items such as cement and labor were assumed to remain stable. Because infrastructure costs account for half of the total five-year scale-up costs, any single percentage point change in infrastructure costs due to changes in the price of cement, labor, or other construction supplies would change the total scale-up costs by half a percentage point.

Teaching staff salaries and housing allowances are preset by the Government of Zambia's Public Service Division and are therefore not expected to change over the five-year period. The additional student recurring costs are also not expected to change significantly. Because student recurring costs account for roughly a third of the overall total costs of expansion, a one percentage point change in the student recurring costs would amount to a one-third percentage point change in the total costs.

Implementation of the training institution expansion will require a significant expansion of currently available funds. The detailed and costed individual school operational plans developed through this assessment can play a significant role in securing funding, since government and donor financial support normally follows rigorous, itemized planning. Already, the operational plans have secured US$ 10 million from the MoH towards implementation in 2008 and in 2009. The immediate funding of the plans by the government and rapid distribution of funds to schools were only possible because the operational plans contained detailed, scheduled and costed steps outlined by cadre and by training institution. Furthermore, if there is a shortage of funds, the operational plans will help the government to understand the relative return of investments in each school, permitting the prioritization of funding according to MoH needs. Private school needs were not included in the national operational scale-up plans. Although the government is open to public-private partnerships to strengthen the private schools and to expand national training capacity, at this time it does not intend to fund infrastructure directly at the private schools.

There were several challenges to training institution scale-up that had to be addressed during the operational planning process to ensure the feasibility of training institution scale-up. First, we learned that planning required significant communication and coordination between numerous MoH departments, professional regulatory bodies, and other ministries. Within the MoH, there are three separate units that oversee the funding, construction, and management of training institutions. Outside of the MoH, the Cabinet Office and the Ministry of Finance control the annual budget for all ministries. The Ministry of Education and the Ministry of Science, Technology and Vocational Training manage the University of Zambia School of Medicine and Evelyn Hone College respectively, two of the three largest multi-program health training institutions in Zambia. The Ministry of Works and Supplies and the National Tender Board must approve and oversee all government construction projects. The General Nursing Council and the Medical Council of Zambia are two professional regulatory bodies outside of government that oversee training quality standards and registration of new graduates prior to their deployment in the public health sector. Improved streamlined coordination between the myriad of involved public and private stakeholders is essential for the effective and timely scale-up of national training infrastructure.

Second, a significant amount of data had to be collected to determine training institution expansion needs. Training institutions in Zambia are diverse and range in complexity, demanding specific, itemized plans per school for successful scale-up management. There are three large training institutions based in Lusaka that offer at least seven highly specialized health training programs in addition to many other non-health related programs. The remaining 36 training institutions operate, at most, three programs and are distributed throughout Zambia. Records on current resources and the operations of each training institution had not been centrally maintained at the MoH, and the granular level of detail required for planning a national expansion of training institutions was not available prior to the field assessments.

Lastly, Zambia is a low-income country with many resource and geographic constraints. Rural training institutions have difficulty attracting and retaining faculty, which is exacerbated by the limited pool of faculty nationwide [12]. Expansion plans for rural schools must address this challenge. There is also limited practical on-site training capacity throughout the country, the expansion of which requires a much broader look at the general health sector infrastructure. MoH capacity constraints also currently limit the speed and volume of hiring and deployment of new graduates, and professional regulatory bodies lack sufficient funding to regulate national training quality and to register new graduates for deployment into the public sector.

There are several limitations to our analysis. First, the actual construction costs may differ from our estimates. We assumed uniform construction costs for the whole country, but actual costs may vary by the location of the training institution due to regional differences in the cost of building materials, labor, and transport. Furthermore, the estimates of costs are not risk-adjusted for market changes other than an assumption of 2% annual inflation, and any fluctuations in building materials or exchange rates will have an impact on construction costs. Second, the timeline for implementation is based on school estimated feasibility and assumes no significant problems with procurement, construction and funding.

## Conclusions

Zambia's model for assessing feasibility and costs can serve as a guide for training scale-up planning in other low-income sub-Saharan African countries. It provides a data-driven and easy-to-use tool for translating national training targets into practical implementation plans. The overall process of designing, executing and distributing the analysis - including target definition and final approval by every training institution - was highly dependent on input from the many stakeholders of health training in Zambia. This ensured buy-in for scale-up plans and support for implementation.

National quality standards were essential to measuring current capacity and to determining expansion needs consistently across cadres and across schools. As such, detailed national training standards for teaching and infrastructure are critical for any country considering national scale-up of training institution capacity.

Expanding national training capacity will require significant investments in infrastructure and faculty. Historic underinvestment in these areas has crippled schools' ability to meet expansion ambitions and quality standards - as evidenced by the overcrowding of dormitories and classrooms and the lack of teaching staff. Significant work remains regarding lobbying for the funds necessary to carry out expansion, but with the increasing commitment of international development agencies to fund pre-service training - including commitments from the United States and Japan to train a quarter of a million health workers over the next ten years. It is hoped that fiscal support for the scale-up of the training institutions in Zambia will be realized on schedule [16,17].

The output of this analysis provided enough details of expansion at each school to give a clear and costed implementation roadmap. The assessment culminated in the creation of the Ministry of Health National Training Operational Plan 2008, which is now being implemented [18]. In 2008, over US$ 16 million in government and partner resources were committed to the implementation of the Training Operational Plan, and over US$ 10 million has been committed in 2009 to date. Once successfully implemented, it is projected that the expansion of training institution capacity will enable the MoH to reach workforce staffing needs by 2022, nearly 20 years earlier than if expansion did not occur [18].

## Competing interests

The authors declare that they have no competing interests.

## Authors' contributions

KS, MK, and ML initiated and managed the research project. AT and JL built the analytical models, assisted in data collection, analyzed the data, and drafted the manuscript. CP and EM assisted with data analysis and drafting of the manuscript. All authors read and approved the final manuscript.
